# Hepatitis C virus (HCV) infection and re-infection among HCV- and HIV/HCV-infected injection drug users

**DOI:** 10.1186/1758-2652-13-S4-P199

**Published:** 2010-11-08

**Authors:** B Conway, A Barrieshee, HK Tossonian, K Wight, S Jassemi, M Tong, E Knight, L Gallagher, F Duncan, S DeVlaming

**Affiliations:** 1 University of British Columbia, Vancouver, Canada; 2 Pender Community Health Centre, Vancouver Coastal Health, Vancouver, Canada

## Background

The possibility of re-infection is often cited as a reason for not initiating treatment in injection drug users (IDUs). Recent observational data suggest that the rate of re-infection may be reduced following spontaneous or treatment-induced virologic clearance, although such data are often retrospective and incomplete.

## Purpose of the study

With this in mind, we have undertaken a systematic, prospective study to evaluate the incidence of HCV viremia in IDUs at risk of new infection.

## Methods

We identified a cohort of IDUs receiving care at the Pender Community Health Centre on Vancouver's Downtown East Side. Potential subjects were identified as either never having been infected with HCV (non-infected arm), having spontaneously cleared the virus (spontaneous arm), or having achieved a sustained virologic response on antiviral treatment (SVR arm). A questionnaire to identify demographics, health status, risk behavior and drug use was administered at baseline and every 6 months, along with blood tests to identify their HCV status.

## Results

A total of 73 subjects met criteria for inclusion in the study: 20 in the non-infected, 30 in the spontaneous and 23 in the SVR arms respectively. Their mean age was 44.7, 17 were female, 9 were HIV-positive. There were no significant differences among the 3 groups with respect to age, ethnicity, source of income, unstable housing and being on opiate maintenance program. Over a mean follow-up period of 10.3 months, 20% of the non-infected group became viremic, as compared to 0% of the other two groups (p=0.04). Injecting drugs in past 30 days (p=0.05), heroin (p=0.015), amphetamines (p=0.05), and combined drugs use (p=0.001) were significantly higher in the non-infected arm compared to SVR arm. There were no significant differences in drug use and risk behavior between non-infected and spontaneous arms.

## Conclusions

Our study shows that viremic HCV infection is more likely to occur in those who have never been previously infected, and that this susceptibility to infection cannot be completely explained by an increase in risk behavior, at least as compared to individuals who have cleared their viremia spontaneously. In addition, HIV co-infection is not a significant factor associated with recurrent HCV viremia. Whether a decreased rate of viremia following SVR relates to some host-related protective factors or is due to a change in IDU-related risk as a result of engagement in the health care system is currently under study.

